# Nuclear factor kappa-light-chain-enhancer of activated B cells gene expression involvement in porcine liver transplant experimental model

**DOI:** 10.1590/acb392724

**Published:** 2024-07-01

**Authors:** Lucas Souto Nacif, Flávio Galvão, Márcia Saldanha Kubrusly, Leonardo Yuri Kasputis Zanini, Paola Espinoza, Daniel Reis Waisberg, Rafael Soares Nunes Pinheiro, Amadeo Batista da Silva, Vinicius Rocha-Santos, Venâncio Avancini Ferreira Alves, Luiz Carneiro-D’Albuquerque, Wellington Andraus

**Affiliations:** 1 Universidade de São Paulo – School of Medicine – Liver and Gastrointestinal Transplant Division – São Paulo (SP), Brazil.

**Keywords:** Liver, Transplantation, Gene Expression, NF-kappaB-Inducing Kinase, Reperfusion Injury

## Abstract

**Purpose:**

Gene expressions of vascular Endothelial Growth Factor Alpha (VEGFa), Nuclear Factor Kappa-Light-Chain-Enhancer of Activated B cells (NFkB) and cytokines could be useful for identifying potential therapeutic targets to alleviate ischemia-reperfusion injury after liver transplantation. Cytokine gene expressions, VEGFa and NFkB were investigated in a preclinical swine model of liver transplantation.

**Methods:**

A total of 12 pigs were used as donors and recipients in liver transplantation without venovenous bypass or aortic clamping. NFkB, IL-6, IL-10, VEGFa and Notch1 gene expression were assessed. These samples were collected in two specific times: group 1 (n= 6) - control, samples were collected before recipient’s total hepatectomy and group 2 - liver transplantation group (n=6), where the samples were collected one hour after graft reperfusion.

**Results:**

Liver transplantation was successfully performed in all recipients. Liver enzymes were elevated in the transplantation group. NFkB gene expression was significantly decreased in the transplantation group in comparison with the control group (0.62±0.19 versus 0.39±0.08; p= 0.016). No difference was observed between groups Interleucine 6 (IL-6), interleucine 10 (IL-10), VEGFa and Notch homolog 1 (Notch1).

**Conclusions:**

In this survey a decreased NFkB gene expression in a porcine model of liver transplantation was observed.

## Introduction

Animal models of liver transplantation are still challenging, due to hemodynamic, anesthetic and postoperative difficulties. Nowadays, few groups are able to perform liver transplantation in swine and keep them alive postoperatively. There are also considerable intraoperative differences between models, as some researchers employ venovenous bypass and others use aortic clamping^1^^-^ 3.

“The use of porcine models in liver transplantation allows for precise characterization of hemodynamic, histological and hepatocellular function changes in porcine models of liver transplantation. The graft function may be affected by the degree of hepatocyte regeneration and of the portal pressure^4^^-^ 8 .

Molecular markers like gene expression have been associated with hepatic regeneration^7^^,^ 9 ^-^ 15. The Vascular Endothelial Growth Factor Alpha (VEGFa) is related to endothelial cell proliferation and hepatic regeneration. The Nuclear Factor Kappa-Light-Chain-Enhancer of Activated B cells (NFkB) is a proinflammatory mediator crucial for maintaining hepatic homeostasis. It contributes to inducing a regenerative response. Furthermore, cytokines gene expression is directly related with hepatocellular proliferation. Its serum levels increase after hepatectomy in healthy livers and induce upregulation of other mitogenic components 7 ^,^ 11 ^,^ 15.

Through a new swine model of liver transplantation without venovenous bypass, we primarily aimed to identify critical factors involved in pathological liver regeneration after ischemia-reperfusion injury. This could be useful for identifying potential therapeutic targets to prevent liver injury in the early events of liver transplantation. There is a lack of surveys assessing for (VEGFa) and (NFkB) and cytokine gene expression in the early period of liver transplantation.

## Methods

### Experimental design

The experiment was approved by the Institutional Animal Use Ethical Committee of Sao Paulo University (Protocol Number 143/16) and involved twelve male swine donors weighing between 20 to 30 kg. The mean weight of donors and recipients were 19 ± 2.75 kg and 25 ± 3.14 kg respectively.

Swine population was divided into two groups: Group 1 - control, in which laboratorial and histologic samples were collected before liver transplantation (n = 6); Group 2 - liver transplantation, in which the same samples were collected 1 hour after graft reperfusion (n = 6).

Blood samples at two different moments (before and one hour after graft reperfusion) were collected and serum levels of hemoglobin, hematocrit, aspartate aminotransferase, alanine aminotransferase, alkaline phosphatase, gamma glutamyltransferase, lactate dehydrogenase, total bilirubin, prothrombin time and creatinine were analyzed.

### Pre-operative care and anesthesia.

The animals were sedated with intramuscular (IM) ketamine (10 mg/kg) and midazolam (0.25 mg/kg) and transferred to the operating room. Anesthesia was induced with intravenous (IV) propofol (5.0 mg/kg) and fentanyl (0.5 mg/kg), followed by endotracheal intubation and mechanical ventilation with positive pressure controlled by volume. Isoflurane 1,5% and IV infusions of pancuronium (0.04 mg/kg/min) and fentanyl (0.075 mg/kg/min) were used for anesthetic maintenance. Antibiotic prophylaxis with ceftriaxone (1.0 g) was administered during anesthesia induction and for two days. The internal jugular vein was dissected and catheterized using a triple lumen catheter for warm crystalloids and colloids solutions (intravenous albumin) infusion and evaluation of central venous pressure. Internal carotid artery was dissected and a catheter was allocated in the artery to monitor invasively arterial pressure and cardiac rhythm.

### Surgical procedure

**Figure 1:**
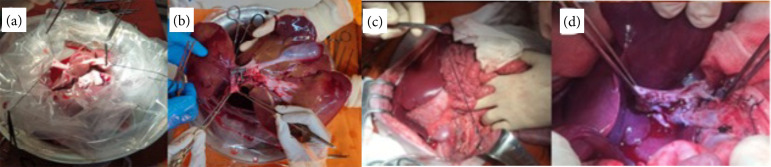
Liver graft during backtable, with the inferior vena cava vein **(a)** and portal vein **(b)** prepared for anastomosis. Aspect of the liver graft after revascularization **(c)** and of the portal and arterial anastomosis **(d)**.

Porcine abdominal cavity was exposed with J laparotomy with allocation of static retractor. The surgical technique for liver transplantation was adapted from the model previously described by Barcelona Group^1^^,^ 3 ^,^ 7. The main steps of our procedure are detailed in Fig. 1. During the follow-up, all animals received IV or IM dipyrone (50 mg/kg) and tramadol (100 mg) twice a day, for two days, and food and water, in accordance with the animal care maintenance protocol. Vital signs and animals’ functionality (food acceptance, mobility, respiration and physiological excretions) were controlled and analyzed, as well as the status of catheters and surgical wounds.

Daily evaluations were conducted on all animals during the postoperative period. Laboratory exams were collected every 3 hours on the 1^st^ day and then every 12 hours until the 5^th^ day post-surgery (end of the experiment). The immunosuppressive regime included corticosteroids bolus (10 mg/kg) in the anhepatic phase and then continued daily (IM) until the 5^th^ post-surgery day.

### Portal vein pressure assessment

During superior mesenteric vein catheterization, an 8 FR polyethylene catheter was placed towards the portal vein, exact localization was confirmed by the surgeon. This catheter was connected to a multiparametric monitor with a dedicated module for venous pressure assessment.

### Euthanasia and discard

The experimental end point was at the 5th postoperative day or if the animals presented clinical deterioration, weight loss greater than 25% of their initial weight or died during the experiment. For the autopsy, anesthesia as previously described. Arterial and venous pressure were measured again and new blood samples and liver biopsy were obtained. Euthanasia was subsequently performed with anesthetic overdose. Animals that did not survive until the end of the experiment were necropsied. All euthanized animals were packed in plastic bags, stored in freezing containers and incinerated in appropriate facilities.

All animal experiments complied with the ARRIVE guidelines and performed in accordance with the National Institutes of Health Guide for the Care and Use of Laboratory Animals. The experiments were approved by the Institutional

Animal Care and Use Committee of São Paulo University (Protocol Number 143/16)

### Biochemical analysis

The blood samples were collected in the donor and in the recipient at the baseline, 1, 3, 6, 12 and 24 hours after reperfusion, and then daily afterwards. The serum levels of hemoglobin, hematocrit, aspartate aminotransferase, alanine aminotransferase, alkaline phosphatase, gamma glutamyltransferase, lactate dehydrogenase, total bilirubin, prothrombin time and creatinine were measured by Elisa. The hepatic tissue was sampled in the donor at the baseline and in the recipient 1 hour after portal reperfusion. Each biopsy was divided into 2 sections, one preserved in 10% formaldehyde for subsequent paraffin inclusion and the other used for gene expression analysis.

### Gene expression analysis

#### RNA extraction, cDNA synthesis and Real Time PCR

Frozen fragments of hepatic tissue were homogenized in TRIzol^TM^ (Invitrogen, Carlsbad, CA, USA) for the extraction of total ribonucleic acid (RNA) according to the manufacturer’s specifications. Total RNA concentration and purity were determined by NanoDrop^TM^ ND-1000 spectrophotometer (Thermo Fisher Scientific, Waltham, MA, USA), using only the RNA samples whose absorbance ratio at 260/280 nm were ≥ 1.8. To analyze RNA integrity, agarose gel electrophoresis was performed to verify the 28S and 18S bands. RNA samples were stored at -80 °C until use.

Total RNA was subjected to reverse transcription for conversion to complementary deoxyribonucleic acid (cDNA) with High-Capacity cDNA Reverse Transcription Kit (Applied Biosystems, CA, USA). The resulting cDNA was stored at -20 °C until use.

Real Time Polymerase Chain Reaction (qRT-PCR) was performed in a StepOnePlus^TM^ thermocycler using TaqMan^®^ Gene Expression Assays (Applied Biosystems, Foster City CA, USA). The probes and primers for genes IL6 (Rn Ss03384604_u1), IL10 (Rn Ss03382372_u1), NFkB (Rn Ss03388575_m1), VEGF (Rn Ss03393993_m1) and Notch 1(Rn Ss03377164_u1), and for the endogenous control, ACTB (Rn Ss03376081_u1), were purchased from Thermo Fisher Scientific (Waltham, MA, USA).

The 2^-∆Ct^ method was used to calculate the expression level of each target gene.

### Statistical analysis

Qualitative and continuous variables were analyzed by Χ² and by Mann-Whitney U tests, respectively, as the Shapiro-Wilk test did not show normal distribution of quantitative variables. Results were expressed as mean ± standard deviation. Statistical significance was set at p < 0.05. Statistical analysis was performed using the GraphPad Prism software 6.0.

## Results

A total of six orthotopic liver transplants were performed according to the current porcine model. The mean cold ischemia time (CIT) and warm ischemia time (WIT) were 120 ± 36.78 minutes and 29 ± 10.28 minutes, respectively. All liver transplants were successfully finished with all animals alive at the end of the first hour after procedure (100% survival); however, a 84% survival after 3 hours, a 66% survival after 6 hours, a 49% survival after 12 hours and a 16% survival after 5 days was observed. All the preoperative, intraoperative and postoperative protocols were strictly followed. The major postoperative complications observed were pulmonary thromboembolism (n = 1), gastric ulcer perforation (n = 1) and biliary fistula (n = 1).

In recipients, the mean cardiac rate at the beginning of the procedure was 99 ± 13.06 bpm. It increased during graft revascularization, reaching 181 ± 41.99 bpm. The mean portal vein pressure was statistically similar before hepatectomy and after the liver transplant (7 ± 1.54 mmHg and 8 ± 0.57 mmHg, respectively).

For the intra- and post-operative exams, it was decided to perform just the analyses of the basal and one hour after the reperfusion period, when all animals were alive and well, without the interference of the surgical complications. The mean aspartate aminotransferase (AST) levels and serum lactate at the beginning of the procedure were 62.4 ± 106.74 U/l and 28.16 ± 19.99 mg/Dl, respectively. They increased to 155.25 ± 61.76 U/l and 96 ± 10.86 mg/dl after reperfusion, respectively. The remaining laboratory data is presented in Table 1.

**Table 1:** Recipients laboratory data (n = 6).

Variable	Time 0	Time 1	P-value
HR, BPM	97 ± 11.07	179 ± 37.81	0.18
MAP, mmHg	78.1 ± 134,1	47.5 ± 201.1	0.002
PVP, mmHg	7 ± 1.54	8 ± 0.57	0.714
CO, L/min	3 ± 0.74	2 ± 1.2	0.114
AST, IU/L	62 ± 106.7	155.25 ± 61.7	0.114
ALT, IU/L	19 ± 6.24	15.6 ± 1.52	0.628
Cr, mg/dl	0.70 ± 0.23	1.25 ± 0.19	0.456
DHL, mg/dl	1726.3 ± 145.1	2922.4 ± 952.9	0.259
FA, IU/L	138.7 ± 39.83	0.17 ± 0.08	0.218
GGT, IU/L	81.8 ± 31.9	26.1. ± 2.9	0.056
BT, mg/dl	0.65 ± 0.26	0.29 ± 0.05	1
BD, mg/dl	0.21 ± 0.12	0.17 ± 0.08	0.218
BIt0, mg/dl	0.43 ± 0.22	0.12 ± 0.05	0.628
Lactate mg/dl	28,16 ± 19.99	96 ± 10.86	0.002
pH	7.345 ± 0.01	7.18 ± 0.02	0.92

Note: Time 0, refers to recipient at the beginning of surgery; Time 1, after revascularization; HR, heart rate; PVP, portal vein pressure; CO, cardiac output; AST, aspartate aminotransferase; CIT, cold ischemia time; WIT, warm ischemia time; Cr, creatinine; DHL, lactate dehydrogenase; FA, alkaline phosphatase; GGT, gamma-glutamyl transferase; BT, total bilirubin; BD, direct bilirubin; BI, indirect bilirubin.

### Genetic expression analysis

**Figure 2:**
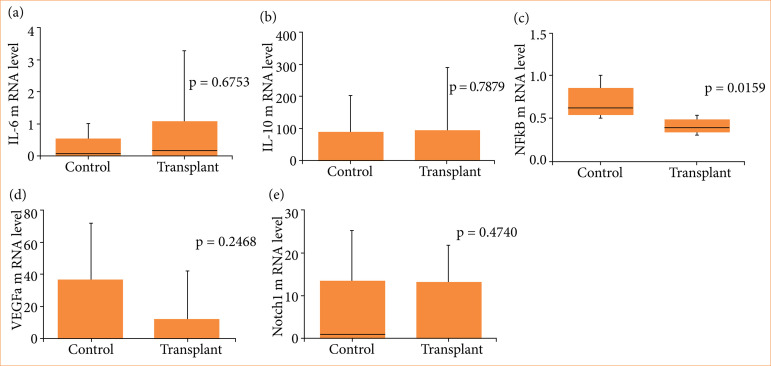
Graphic representation (boxplot) of gene expression of mRNA levels. **(a)** IL-6 gene. **(b)** IL-10 gene. **(c)** NFkB gene. **(d)** VEGFa gene. **(e)** Notch1 gene.

NFkB gene expression was significantly reduced in transplanted animals as compared to the control group (0.62 ± 0.19 versus 0.39 ± 0.08, respectively p = 0.0159). However, there was no difference between the study groups for IL-6, IL-10, VEGFa and Notch1 gene expression. (Table 2 and Fig. 2).

**Table 2:** Comparative values of gene expression (relative mRNA level).

Gene	Control (n = 6)	Transplant (n = 6)	p-value
IL-6	0.07 ± 0.39	0.16 ± 1.28	0.6753
IL-10	3.33 ± 81.09	2.58 ± 116,5	0.7879
NFkB	0.62 ± 0.19	0.29 ± 0.08	0.0159
VEGFa	0.81 ± 31.8	0.55 ± 16.90	0.2468
Notch1	1.11 ± 9.96	0.58 ± 8.89	0.4740

## Discussion

Models of porcine liver transplantation are very useful for pre-clinical analysis of innovative and complex procedures. The physiology and anatomy of these animals resemble those of humans. Furthermore, they have favorable hemodynamic conditions and homogeneity among their species 4 ^-^ 9. The improvement in surgical technique and postoperative follow-up protocols in this model allowed the development of clinical and experimental liver transplantation models. In the current model, for the first time, the gene expression of VEGFa and the NFkB, Notch1, IL-6 and IL-10 genes were analyzed in the early phase of liver transplantation. However, only the expression of the NFkB gene in the transplanted animals statistically decreased compared to the control group.

The expression of the NFkB gene is triggered by organic stress processes^15^. NFκB activation results in the initiation of inflammatory, immune and wound-healing responses and in the clearance of pathogens. The production of cytokines involved in liver injury conditions leads not only to the activation of signaling cascades related to hepatocellular injury and Kupffer cell activation, but also to the induction of pro-regenerative substances, important for the restoration of liver function and volume, such as Stat3 and NFkB factors^14^^-^ 20.

The imbalance of these hepatocellular proliferation reactions can lead to pathological processes related to both liver cirrhosis and hepatocellular regeneration after transplantation.

Phavalan et al.^20^ described three moments of regeneration: initiation, proliferation and inhibition. Considering the initiation components, cytokines play a central role in the outcome of the signaling cascades, with IL-6, TNF-α, IFN-γ, lymphotoxin-beta (LT-β) and nitric oxide (NO) being responsible for a greater cellular sensitization to growth factors and for increased production of NFkB by oval liver cells^20^. Post-translational modification of proteins such as NFkB is a key element in making hepatocytes more susceptible to growth factors at the early period of liver regeneration^22^^,^ 23.

In the present study, there is a significantly lower NFkB expression in the transplanted group. It could be caused by the use of the glucocorticoid for immunosuppression that is recognized for cause reduction of NFkB expression^22^. In fact, the activation of the noncanonical NFkB signaling pathway is often observed in a number of liver diseases. Therefore, the modulation of this pathway might be a promising therapeutic strategy to reduce liver inflammation. Other drugs could reduce NFkB expression, including blockade of TNF, interleukin 1, and IL-6 mediators. The application of this concept could assist in regulating hepatic regeneration..

Despite the biological plausibility of the decreased NFkB gene expression in glucocorticoids used in transplant group, the results found in this experimental model must be confirmed in studies with larger samples. The molecular components that are related to liver regeneration are not completely understood, but many complex biomolecular pathways are believed to participate in the process.

This study had some limitations since it is an experimental model with a small animal sample. On the 5th day following surgery, there was a higher mortality rate, suggesting a potential bias not only from numerical factors but also from intra- and postsurgical physical effects.

However, the molecular findings of our study may have contributed to the development of other preclinical models. The aim was to search for drugs that modulate the hepatocellular regenerative capacity, which may enable smaller liver grafts in living donor liver transplantation.. This could reduce the need of large donor hepatectomies.

## Conclusion

A decreased expression of the NFkB gene in a porcine experimental model of liver transplantation was observed. These findings may allow the development of novel therapeutics for the NFkB gene expression modulation.

## Data Availability

Data will be available upon request

## References

[1] Hessheimer AJ, Fondevila C, Taurá P, Muñoz J, Sánchez O, Fuster J, Rimola A, García-Valdecasas JC (2011). Decompression of the portal bed and twice-baseline portal inflow are necessary for the functional recovery of a “small-for-size” graft. Ann Surg.

[2] Kothary PC, Kokudo N, Eckhauser FE, DelValle J, Raper SE (1995). Preferential suppression of insulin-stimulated proliferation of cultured hepatocytes by somatostatin: evidence for receptor-mediated growth regulation. J Cell Biochem.

[3] Hessheimer AJ, Escobar B, Muñoz J, Flores E, Gracia-Sancho J, Taurá P, Fuster J, Rimola A, García-Valdecasas JC, Fondevila C (2014). Somatostatin therapy protects porcine livers in small-for-size liver transplantation. Am J Transplant.

[4] Dahm F, Georgiev P, Clavien PA (2005). Small-for-size syndrome after partial liver transplantation: definition, mechanisms of disease and clinical implications. Am J Transplant.

[5] Imura S, Shimada M, Ikegami T, Morine Y, Kanemura H (2008). Strategies for improving the outcomes of small-for-size grafts in adult-to-adult living-donor liver transplantation. J Hepatobiliary Pancreat Surg.

[6] Heaton N. (2003). Small-for-size liver syndrome after auxiliary and split liver transplantation: donor selection. Liver Transpl.

[7] Fondevila C, Hessheimer AJ, Taurá P, Sánchez O, Calatayud D, de Riva, Muñoz J, Fuster J, Rimola A, GarcíaValdecasas JC (2010). Portal hyperperfusion: mechanism of injury and stimulus for regeneration in porcine small-for-size transplantation. Liver Transpl.

[8] Zhong RF, Kai Y, He L, Yang L, Bunzendahl H, Brenner DA, Lemasters JJ (2006). Liver regeneration is suppressed in small-for-size liver grafts after transplantation: involvement of c-Jun N-terminal kinase, cyclin D1, and defective energy supply. Transplantation.

[9] Ninomiya M, Shirabe K, Terashi T, Ijichi H, Yonemura Y, Harada N, Soejima Y, Taketomi A, Shimada M, Maehara Y (2010). Deceleration of regenerative response improves the outcome of rat with massive hepatectomy. Am J Transplant.

[10] Belghiti J, Liddo G, Raut V, Zappa M, Dokmak S, Vilgrain V, Dondéro F (2012). Inherent limitations” in donors: control matched study of consequences following a right hepatectomy for living donation and benign liver lesions. Ann Surg.

[11] Ross MA, Sander CM, Kleeb TB, Watkins SC, Stolz DB (2001). Spatiotemporal expression of angiogenesis growth factor receptors during the revascularization of regenerating rat liver. Hepatology.

[12] Kamel IR, Erbay N, Warmbrand G, Kruskal JB, Pomfret EA, Raptopoulos V (2003). Liver regeneration after living adult right lobe transplantation. Abdom Imaging.

[13] Haga J, Shimazu M, Wakabayashi G, Tanabe M, Kawachi S, Fuchimoto Y, Hoshino K, Morikawa Y, Kitajima M, Kitagawa Y (2008). Liver regeneration in donors and adult recipients after living donor liver transplantation. Liver Transpl.

[14] Canedo BF, Galvao FH, Ducatti L, Nacif LS, Catanozi S, Soler WV, Chaib E, D’Albuquerque LA, Andraus W (2015). Liver Autotransplantation in Pigs without Venovenous Bypass: A Simplified Model using a Supraceliac Aorta Cross-Clamping Maneuver. Ann Transplant.

[15] Gilmore T. (1999). The Rel/NF-κB signal transduction pathway: introduction. Oncogene.

[16] Taub R. (1996). Liver regeneration in health and disease. Clin Lab Med.

[17] Nacif LS, Ferreira AO, Maria DA, Kubrusly MS, Molan N, Chaib MS, D’Albuquerque LC, Andraus W (2015). Which is the best route of administration for cell therapy in an experimental model of small-for size syndrome in rats?. Acta Cir Bras.

[18] Fernandes MR, Nacif LS, Alvarez PSE, Pinheiro RS, Rocha-Santos V, de Martino RB, Waisberg DR, Macedo RA, Ducatti L, de Paiva LB, Galvão FHF, Andraus W, Carneiro-D Albuquerque, Small-for-Size Syndrome: Systemic Review in a Porcine Experimental Model (2022). Small-for-Size Syndrome: Systemic Review in a Porcine Experimental Model. Transplant Proc.

[19] Nacif LS, Alvarez PSE, Pinheiro RS, Da Silva AB, Fernandes MR, Santos JPC, Ernani L, Rocha-Santos V, De Martino RB, Waisberg DR, Macedo RA, Ducatti L, Haddad L, Galvão FHF, Andraus W, Carneiro-D L (2022). Experimental Clinical Model of Liver Transplantation in Large White Pigs Without Venovenous Bypass: Pre-, Intra-, and Maintenance Care. Transplant Proc.

[20] Pahlavan PS, Feldmann RE, Zavos C, Kountouras J (2006). Prometheus’ challenge: molecular, cellular and systemic aspects of liver regeneration. J Surg Res.

[21] Chen Q, Lu X, Zhang X (2021). Noncanonical NF-κB Signaling Pathway in Liver Diseases. J Clin Transl Hepatol.

[22] Molinero LL, Alegre ML (2012). Role of T cell-nuclear factor κB in transplantation. Transplant Rev (Orlando).

[23] Dashti-Khavidaki S, Saidi R, Lu H (202). Current status of glucocorticoid usage in solid organ transplantation. World J Transplant.

